# Is frailty a stable predictor of mortality across time? Evidence from the Cognitive Function and Ageing Studies

**DOI:** 10.1093/ageing/afy077

**Published:** 2018-06-16

**Authors:** Andria Mousa, George M Savva, Arnold Mitnitski, Kenneth Rockwood, Carol Jagger, Carol Brayne, Fiona E Matthews

**Affiliations:** 1 Department of Infectious Disease Epidemiology, School of Public Health, Imperial College London, London, UK; 2 School of Health Sciences, University of East Anglia, Norwich, UK; 3 Department of Medicine, Dalhousie University, Nova Scotia, Canada; 4 Division of Geriatric Medicine, Department of Medicine, Dalhousie University, Nova Scotia, Canada; 5 Institute of Health and Society, Newcastle University Institute for Ageing, Newcastle University, Newcastle, UK; 6 Department of Public Health and Primary Care, Cambridge Institute of Public Health, Cambridge, UK; 7 MRC Biostatistics Unit, Cambridge University, Cambridge, UK

**Keywords:** frailty, mortality, frailty index, Cognitive Function and Ageing Study (CFAS), older people

## Abstract

**Background:**

age-specific mortality reduction has been accompanied by a decrease in the prevalence of some diseases and an increase in others. Whether populations are becoming ‘healthier’ depends on which aspect of health is being considered. Frailty has been proposed as an integrative measure to quantify health status.

**Objective:**

to investigate changes in the near-term lethality of frailty before and after a 20-year interval using the frailty index (FI), a summary of age-related health deficit accumulation.

**Design:**

baseline data from the Cognitive Function and Ageing Studies (CFAS) in 1991 (*n* = 7,635) and 2011 (*n* = 7,762).

**Setting:**

three geographically distinct UK centres (Newcastle, Cambridgeshire and Nottingham).

**Subjects:**

individuals aged 65 and over (both institutionalised and community-living).

**Methods:**

a 30-item frailty score was used, which includes morbidities, risk factors and subjective measures of disability. Missing items were imputed using multiple imputations by chained equations. Binomial regression was used to investigate the relationship between frailty, age, sex and cohort. Two-year mortality was modelled using logistic regression.

**Results:**

mean frailty was slightly higher in CFAS II (0.19, 95% confidence interval (CI): 0.19–0.20) than CFAS I (0.18, 95% CI: 0.17–0.18). Two-year mortality in CFAS I was higher than in CFAS II (odds ratio (OR) = 1.16, 95% CI: 1.03–1.30). The association between frailty and 2-year mortality was non-linear with an OR of ~1.6 for each 0.10 increment in the FI.

**Conclusions:**

the relationship between frailty and mortality did not significantly differ across the studies. Severe frailty as an indicator of mortality is shown to be a stable construct.

Recent advancements in health have led to reductions in mortality and increased life expectancy in both high- and low-income countries. Consequently, the prevalence of age-related chronic diseases, such as diabetes mellitus, ischaemic heart disease and liver disease, has increased [**1, 2**]. Even so, reductions have been observed in cognitive impairment and functional disability [3]. Deciding whether decreases in mortality are a result of health improvements therefore depends on which aspect of health is being measured. The relationship between different health conditions and mortality is complex, so characterising a population’s health in terms of each condition is challenging. As an integrative concept, frailty in the clinical sense brings together different health components, including disability and cognition [4].

Frailty is characterised as the loss of reserves leading to a state of increased vulnerability for adverse health outcomes [4–8]. One approach to measuring frailty is by an index of ‘deficit accumulation’ [5, 9]. The frailty index (FI) can be a better predictor of mortality than chronological age [10–12], and relative to other frailty instruments, has a high predictive validity for a number of negative outcomes [13, **14**].

Frailty measures are being increasingly adopted within clinical settings, including primary and social care. In the UK, the electronic frailty index (eFI) consisting of routinely collected primary care electronic health record data has been validated against mortality, hospitalisation and nursing home admission using data from 931,541 patients [15]. In general, the FI is a useful indicator of needs, which include hospital discharge, institutionalisation or continuing care [**16**]. Despite the established association between frailty and adverse outcomes, as well as the increase in health service use [17], quality of care for those with high frailty scores is often inadequate with unmet healthcare needs [**18, 19**]. The FI has a policy relevance which needs to be underpinned by thorough understanding of its performance across time.

Large longitudinal studies have studied frailty and its association with survival up to 15 years [20–22]. Most frailty research has investigated long-term survival and mortality over extended periods [23]. Clinical utility for the index is likely to be more useful over shorter periods, but limited research has examined how the relationship between frailty and short-term mortality may have changed [23]. Using a 30-item FI based on variables collected in the Medical Research Council Cognitive Function and Ageing Study, we examine whether frailty provides a stable indicator of mortality risk across decades, therefore validating its status as a robust marker of need. Alternatively, if the performance of frailty changes this may be because the variables making it up now have different implications.

## Methods

### Study design

The Cognitive Function and Ageing Studies (CFAS) are population-based longitudinal studies of ageing which examine risk factors and health outcomes in the older UK population. Recruitment for CFAS I began in 1991 and CFAS II was initiated in 2008. Full details of the methods have been published elsewhere [24, 25] and are explained in brief here.

In three geographical sites (two urban, Newcastle and Nottingham, and one rural, Cambridgeshire), using the same sampling methods (National Health Service Primary Care lists), random samples of people aged 65 and over were drawn (CFAS I, *n* = 7,635 and CFAS II, *n* = 7,762 at baseline). All participants were flagged at the Office for National Statistics for routine death notification to determine mortality up to 2 years from initial interview.

### Construction of the CFAS frailty index

The CFAS-FI is composed of 30 items—or deficits—and subject to best practice in determining the FI, these items accumulate with age, influence vulnerability to adverse outcomes, and are neither very rare nor very common in the population [9]. Each deficit is weighted equally and is scored based on its presence or absence. The FI is expressed as a proportion of deficits present out of the total answered. The deficits (Appendix A in the Supplementary data, available at *Age and Ageing* online), were measured identically in CFAS I and II and include items across a range of health domains.

Missing frailty data were partly completed using informant assessment interviews at baseline, from relatives or friends. The informant’s response was only taken into account for those with missing items, and where informants were certain about the subject’s condition.

### Statistical analysis methods

Binomial regression was used to investigate the relationship between frailty, age and sex initially stratified by, and then adjusted for, cohort. To investigate the relationship between frailty and mortality risk, a logistic regression analysis with death at 2 years was undertaken, adjusting for age, sex and cohort. To model a non-linear association between frailty and mortality, a quadratic term was included in addition to the main effect of frailty. The presence of any potential interactions between frailty, study, age and sex was tested. Receiver operating characteristic curve analysis was used to calculate the area under the curve (AUC) for the mortality models. Sensitivity analyses were undertaken including (a) Cox regression, taking into account time to death up to 2 years, (b) removing three items (medicated hypertension, diabetes and thyroid problems), which are now more readily diagnosed and (c) removing two items (cognitive impairment and depression), which are markers of non-participation.

Non-response weights, calculated using inverse probability weighting, were used throughout to adjust for initial non-response (20% in CFAS I and 44% in CFAS II) by sex, age, living in care and deprivation [24]. Missing data in the FI (8.4% of participants) were investigated initially by excluding all individuals with missing data (complete-case analysis), followed by pro-rata calculation for those with only one missing deficit, together with missing data imputation using chained equations (MICE). Ten iterations were used to impute missing data. Each frailty item was imputed separately before calculating the score. Observed hearing had the fewest missing values (1.0%). Deficits with the most missing values were difficulty to get on a bus (3.7%) and self-reported health (3.5%). The regression used for the imputation adjusted for the response of all other items, as well as age, sex and study. Here, we present the results of the imputed analysis as the main analysis (results of all methods are tabulated in Appendix B, in the Supplementary data, available at *Age and Ageing* online).

## Results

### Sample characteristics

Both cohorts included more women than men (CFAS I: 61%, CFAS II: 56%), and by design, the median age in the population was 75. The submaximal limit to frailty incorporating 99% of the subjects was 0.57 (maximal limit = 0.73) in both studies. Scores on the FI were positively skewed with a median of 0.13 in CFAS I and 0.17 in CFAS II (Appendix C in the Supplementary data, available at *Age and Ageing* online). Mean frailty was slightly higher in CFAS II (0.19, 95% confidence interval (CI): 0.19–0.20) than CFAS I (0.18, 95% CI :0.17–0.18), with a relative increase of 8.4%. Appendix A, in the Supplementary data, available at *Age and Ageing* online gives the crude, unadjusted proportions for each of the deficits. The highest relative increase across time was observed for medicated hypertension (24%). Cognitive impairment and transient ischaemic attack had the highest relative decrease (7% and 6%, respectively).

### Age and sex-specific frailty

In the unadjusted analysis women had higher scores than men in both studies (Appendix D in the Supplementary data, available at *Age and Ageing* online). Both genders had slightly higher frailty scores (0.5 more deficits on average) in CFAS II than CFAS I (difference = 0.015, 95% CI: 0.01–0.02). The mean frailty score for men was 0.15 in CFAS I and 0.16 in CFAS II and for women it was 0.20 and 0.21, respectively. Median frailty indices were lower because of the skewed distribution (Appendix D in the Supplementary data, available at *Age and Ageing* online).

**Figure 1.**
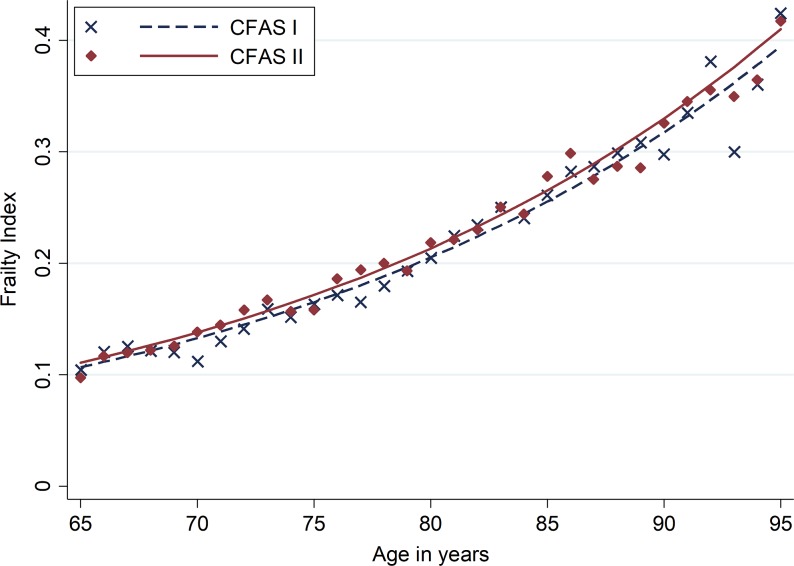
The relationship between age and the frailty in Cambridgeshire, Newcastle and Nottingham, UK, 1991 (CFAS I) and 2011 (CFAS II). The frailty index is shown as a proportion of deficits at baseline. The solid and dotted lines show the predicted frailty as derived from the binomial model. The symbols denote the observed means of frailty at each age, calculated from the imputed dataset.

The relationship between the FI and age by study is shown in Figure 1 and Appendix E in the Supplementary data, available at *Age and Ageing* online, which takes into account gender differences. Women had more deficits than men (relative risk (RR) = 1.21, 95% CI: 1.18–1.24) and there was a steady increase in frailty with age (RR = 1.23 for each 5-year increase, 95% CI: 1.22–1.24). There was no evidence of an interaction effect between age and sex (*P* = 0.44). When modelling the two studies separately, the relationship between age and sex on frailty was similar.

### Frailty index and mortality

Two-year mortality in CFAS II was significantly lower than in CFAS I (odds ratio (OR) = 0.86, 95% CI: 0.77–0.97). Within 2 years, 819 participants (10.7%) died in CFAS I whereas 643 (8.3%) died in CFAS II. In CFAS I, the unadjusted odds of dying for men was 1.36 times that of women (95%CI:1.18–1.58). Death in men declined (from 12.9% in CFAS I to 9.1% in CFAS II) but in women it remained constant (9.8% vs. 10.0%). Hence, the difference in 2-year mortality between men and women was no longer significant in CFAS II (OR = 0.90, 95% CI: 0.76–1.07).

**Figure 2.**
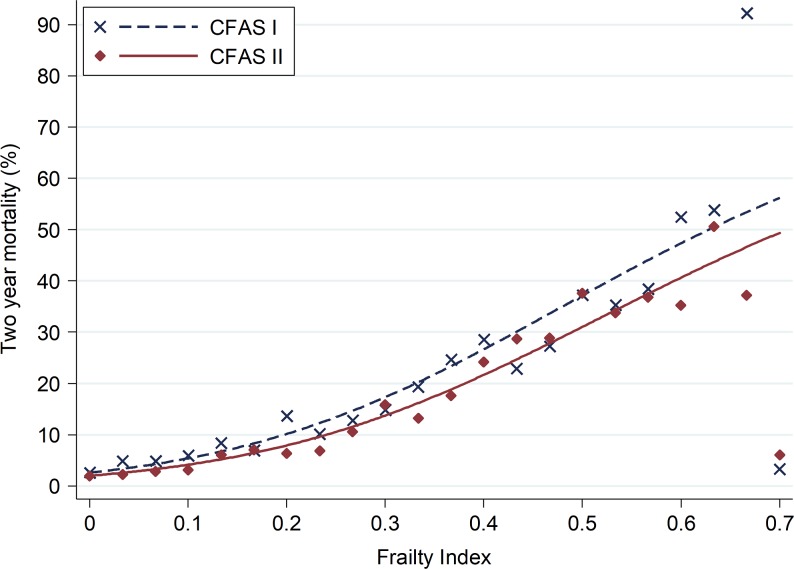
Crude 2-year mortality (%) by frailty index in CFAS I and II. The solid and dotted lines show the predicted frailty as derived by the logistic regression model and the symbols denote the means of mortality for each frailty score as a proportion of deficits.

The FI was a significant predictor of mortality after adjustment for study (Appendix F—Model A in the Supplementary data, available at *Age and Ageing* online). Figure 2 shows 2-year mortality increasing with increasing frailty. For the less frail, 2-year mortality was similar in CFAS I and II but for the more frail, mortality decreased in CFAS II. For instance, the predicted probability of dying within 2 years for a participant scoring 0.1 on the FI is 1.2% lower in CFAS II than in CFAS I (4.1% vs. 5.4%, OR = 1.32). However, for a FI of 0.5 (OR = 1.20), the predicted probability of death within 2 years was 37.0% in CFAS I and 30.7% in CFAS II. Mortality means at high frailty levels were calculated from a small number of individuals as frailty scores of over 0.6 were extremely rare (<1% of the sample), explaining the high variability in observed mortality for high frailty scores (Figure 2).

The relationship between frailty and mortality was adjusted for age, sex and study (Appendix F—Model B in the Supplementary data, available at *Age and Ageing* online). The adjusted OR for the FI was ~1.6 for each 0.1 increase in the FI. The odds of 2-year mortality for men was almost 80% greater than for women (OR = 1.78, 95% CI: 1.56–2.03). For each 5-year increase in age, the odds of 2-year mortality increased by half (OR = 1.52, 95% CI:1.45–1.59). Accounting for frailty, age and sex, mortality in CFAS I was considerably higher than CFAS II (OR = 1.48, 95% CI: 1.31–1.67). Interactions were found between the effects of age and study, and sex and study on mortality. These were clinically small but statistically significant and therefore included in the final model for completeness (Appendix B in the Supplementary data, available at *Age and Ageing* online). The AUC for the imputed analysis was slightly higher when compared with the AUC for the complete-case analysis and that including response to 29 items. After adjustment of all variables and significant interactions, the effect of frailty on mortality was higher in the imputed analysis than using the other methods (Appendix B in the Supplementary data, available at *Age and Ageing* online). The predicted probabilities derived from the final model are shown in Appendices G and H, in the Supplementary data, available at *Age and Ageing* online. In this model, the odds of 2-year mortality were over four times higher (OR = 4.34, 95% CI: 3.23–5.45) in those with a FI of 0.30 compared with those with a FI of zero (Appendix F—Final model in the Supplementary data, available at *Age and Ageing* online).

The age and sex-adjusted relationship of frailty and mortality were also tested in an analysis stratified by study (Table 1). The difference in fatality of frailty between the cohorts was not statistically significant (OR for interaction = 1.61, 95% CI: 0.68–3.83, *P* = 0.28; Table 1), indicating that the relationship between mortality and frailty has not significantly changed over time. In CFAS II, the relationship between mortality and frailty was non-linear, therefore comparisons between each increment of additional frailty between the studies is influenced by the non-linearity. In CFAS I, each 0.10 increase in the FI had an OR of 1.57, but for CFAS II the OR was initially higher (1.72) reducing to 1.55 with increasing frailty. The FI had a high discrimination for predicting death up to 2 years, with an AUC of 0.72 in CFAS I and 0.77 for CFAS II (Appendix I in the Supplementary data, available at *Age and Ageing* online).

**Table 1.** The relationship between frailty and mortality in an analysis stratified by study. Separate models were run for CFAS I and II. Both models included the non-linear effect of frailty and were adjusted for age and sex

	Effect of a 0.1 increase in FI		Effect compared to FI = 0	
Frailty index (FI)	Ref. FI	Odds ratio (95% CI)	Ref. FI	Odds ratio (95% CI)
CFAS I				
0.1	0	1.57 (1.31, 1.83)	0	1.57 (1.31, 1.83)
0.2	0.1	1.57 (1.41, 1.73)	0	2.47 (1.82, 3.11)
0.3	0.2	1.57 (1.48, 1.66)	0	3.87 (2.69, 5.05)
0.4	0.3	1.57 (1.46, 1.69)	0	6.08 (4.25, 7.92)
0.5	0.4	1.57 (1.37, 1.77)	0	9.55 (6.81, 12.30)
0.6	0.5	1.57 (1.27, 1.87)	0	15.00 (9.91, 20.09)
0.7	0.6	1.57 (1.17, 1.97)	0	23.56 (11.49, 35.63)
CFAS II				
0.1	0	1.72 (1.37, 2.07)	0	1.72 (1.37, 2.07)
0.2	0.1	1.69 (1.46, 1.91)	0	2.90 (1.93, 3.87)
0.3	0.2	1.66 (1.53, 1.79)	0	4.81 (2.89, 6.73)
0.4	0.3	1.63 (1.51, 1.75)	0	7.84 (4.66, 11.02)
0.5	0.4	1.60 (1.39, 1.81)	0	12.56 (7.78, 17.34)
0.6	0.5	1.57 (1.26, 1.89)	0	19.77 (12.05, 27.48)
0.7	0.6	1.55 (1.12, 1.97)	0	30.55 (14.55, 46.59)

Sensitivity analysis using Cox regression revealed no change to the conclusions (Appendix J in the Supplementary data, available at *Age and Ageing* online). Removing hypertension, diabetes and thyroid items from the index resulted in lower frailty indices in CFAS II, which seem to explain the cohort difference in frailty. Conversely, removing cognitive impairment and depression from the index resulted in higher scores in CFAS II. However, no substantial differences were observed in the mortality models’ coefficients for either of the sensitivity analyses (Appendix J in the Supplementary data, available at *Age and Ageing* online).

## Discussion

Our key findings from this comparison of frailty across two decades are that, despite a slight increase in the frailty of the population and a marked decrease in the mortality of the general population aged 65 and over in three geographical regions in the UK, the relationship between frailty and 2-year mortality has been relatively stable. This complex relationship may be, at least partly, accounted for by a reduction of mortality at lower levels of frailty and improved diagnosis.

### Strengths and weaknesses

One limitation of the study is that frailty does not reflect a trajectory as it is measured only at one timepoint, with different cohorts providing age-specific cross-sectional associations. The use of longitudinal studies to investigate temporal changes in frailty with respect to adverse outcomes may help strengthen the conclusions. For example, in the Canadian National Population Health Survey, analyses of longitudinal data showed a greater rate of change in frailty scores over 16 years than was inferred from cross-sectional data [26]. In addition, response rates were much lower in CFAS II than CFAS I, a bias partly addressed by the inverse probability weights. Unmeasured factors influencing cohort participation may bias our frailty estimates. Cognitive impairment and depression, however, did not affect the conclusions as indicated by the sensitivity analysis on the mortality effects.

The non-specific nature of the FI has led to some criticism but it reflects the non-specific and complex nature of ageing itself [**27**]. Despite being represented by a single deficit, consequences of serious health conditions are reflected in additional deficits. In addition, the deficits used were self-reported with the exception of mobility, eyesight, hearing, and cognitive impairment, which were test-based. There is some evidence to suggest that a FI is more robust with the inclusion of both test-based and self-reported deficits [28].

Large sample sizes in the CFAS studies allow for increased power to detect differences, and the MICE method preserves this statistical power by using all available data and producing less biased estimates [29]. A study by McCaul *et al.* [30], found that imputed frailty estimates were ~10% higher than complete-case analysis estimates. Similarly, we found that the adjusted frailty effect on mortality was higher in the imputed analysis than the complete-case analysis. This indicates that missingness of frailty may be associated with higher mortality levels which is consistent with our understanding of missingness in longitudinal studies. However, this difference was not significant in this study.

### Findings

Our findings agree with previous reports that women have higher frailty scores but lower mortality than men [22, 31, 32, **33**]. This may be explained by the concepts of male ‘fitness-frailty pleiotropy’ and female ‘fertility-frailty pleiotropy’ which result in lower physiological reserves in men. It is hypothesised that the threshold for system failure at old age in men is low as a result of better physiological functioning during youth, but in women this threshold has increased by limiting childbearing [34]. Similar to most studies [9, 35, 36], the maximum FI was ~0.7 suggesting that surviving beyond that is unlikely. Differences in submaximal and maximal limits have been reported, supporting the theory that women have higher physiological reserves than men [20, 36–38].

Despite a decrease in the absolute 2-year mortality risk for a given level of frailty, the RR of mortality has remained unchanged in CFAS II. These findings contrast with a Swedish study that compared 70-year olds born in 1900 with those born in 1930; in the latter, the long-term lethality of frailty declined [23]. This could reflect period or country effects, and particularly differences in healthcare systems or a longer interval between cohorts. Overall, the estimate of the association of frailty with mortality is consistent with those from other cohorts [8], with an OR of ~1.6 for every 0.1 increment in the FI. Higher frailty indices in CFAS II were mostly attributable to increases in the proportion of those with disabilities and morbidities such as diabetes, hypertension, meningitis, arthritis and thyroid problems (Appendix A in the Supplementary data, available at *Age and Ageing* online). Changes in practice that occurred between the two cohorts such as the Quality and Outcomes Framework for primary care introduced in 2004 may have led to an increase in the reporting of conditions such as diabetes and hypertension, particularly at milder stages. Frailty in the CFAS I population may have been relatively underestimated, partly explaining the observed decrease in absolute mortality, despite the apparent increase in frailty. It may also be that only conditions with an early onset have increased in prevalence whereas those closer to death have decreased or remained the same. Moreover, a substantial proportion of the variability in mortality does not seem to be explained by frailty, sex, age or cohort. Other factors such as sociodemographic and behavioural determinants may be influencing frailty. A model adjusting for such factors may improve prediction of both frailty and mortality.

## Conclusions

Our results, from two similar population-based studies carried out 20 years apart, reveal that despite lower mortality in recent years, older populations have a higher FI and the relationship of frailty with mortality is essentially unchanged for the two generational cohorts. It is likely that changes in reporting and diagnostic practice have increased the prevalence of lesser degrees of frailty, but despite this the relationship between frailty and mortality has remained stable over two decades. This has implications for both policy planning and clinical practice. Although the stable frailty–mortality relationship might reflect when and where our study was done, it is worth considering why, unlike the Swedish study, the RR for mortality in relation to frailty did not decline. One possible explanation is that an interventional service response sufficient to meet needs is required [**39**], something that emerging experience with the eFI [15] along with further investigation in contemporary cohorts will be able to clarify.

**Key points**

- The FI of an ageing population allows relationships between mortality and a unified measure of health to be examined.
- A 12.3% relative decrease in mortality and an 8.4% relative increase in frailty were observed in two cohorts set 20 years apart.
- The relationship between frailty and mortality has not changed, despite changes in occurrence and death rates over time.
- This complex relationship may be accounted for by a reduction of mortality at lower levels of frailty and improved diagnosis.
- A measure of frailty is a stable tool through time that can be used to indicate individuals at higher risk of mortality.

## Supplementary Material

Supplementary Data

Supplementary Data
