# Supplementary material for: Is frailty a stable predictor of mortality across time? Evidence from the Cognitive Function and Ageing Studies
Source: Age Ageing. 2018 Jun 16;47(5):721–7. doi: 10.1093/ageing/afy077 (PMC6108394; doi:10.1093/ageing/afy077)
Supplement: Supplementary Data [file afy077_aa-17-1121-file003.docx]

**SUPPLEMENTARY DATA**

**Appendix 1- Prevalence (and 95% CI) of all deficits (n=30) included in the frailty index by study as a percentage.**

|  | **Deficit** |  | **Prevalence % (95% CI)** | | | | | |  | **% Relative change** |  |
| --- | --- | --- | --- | --- | --- | --- | --- | --- | --- | --- | --- |
|  |  |  | **CFAS I** | | | **CFAS II** | | |  |  |  |
|  |  |  |  |  |  |  |  |  |  |  |  |
| **Medical conditions** | |  |  |  |  |  |  |  |  |  |  |
|  | Angina |  | 17·2 | (16·3, | 18·0) | 16·0 | (15.2, | 16.9) |  | -1.2 |  |
|  | Arthritis |  | 53.0 | (51.8, | 54.1) | 54.1 | (52.9, | 55.2) |  | 1.1 |  |
|  | Depressionᵅ |  | 10.4 | (9.7, | 11.1) | 7.4 | (6.8, | 8.0) |  | -3.0 |  |
|  | Diabetes mellitus |  | 6.3 | (5.7, | 6.8) | 14.5 | (13.6, | 15.3) |  | 8.2 |  |
|  | Epileptic Fits |  | 2.1 | (1.8, | 2.5) | 2.2 | (1.9, | 2.6) |  | 0.1 |  |
|  | Head injury |  | 12.3 | (11.6, | 13.1) | 11.3 | (10.5, | 12.0) |  | -1.0 |  |
|  | Heart attack |  | 10.2 | (9.5, | 10.8) | 11.4 | (10.7, | 12.2) |  | 1.2 |  |
|  | Intermittent claudication |  | 4.3 | (3.8, | 4.8) | 2.8 | (2.4, | 3.2) |  | -1.5 |  |
|  | Medicated hypertension |  | 26.4 | (25.4, | 27.4) | 50.2 | (49.0, | 51.3) |  | 23.8 |  |
|  | Meningitis or encephalitis |  | 0.8 | (0.6, | 1.0) | 2.0 | (1.7, | 2.3) |  | 1.2 |  |
|  | Parkinson's disease |  | 1.1 | (0.8, | 1.3) | 0.9 | (0.7, | 1.1) |  | -0.2 |  |
|  | Peptic ulcers |  | 10.2 | (9.5, | 10.9) | 8.4 | (7.8, | 9.1) |  | -1.8 |  |
|  | Stroke |  | 7.9 | (7.3, | 8.5) | 8.6 | (7.9, | 9.3) |  | 0.7 |  |
|  | Thyroid problems |  | 7.3 | (6.7, | 7.9) | 12.9 | (12.1, | 13.6) |  | 5.6 |  |
|  | Transient ischaemic attack |  | 14.7 | (13.9, | 15.5) | 8.5 | (7.8, | 9.1) |  | -6.2 |  |
|  |  |  |  |  |  |  |  |  |  |  |  |
| **Other conditions** | |  |  |  |  |  |  |  |  |  |  |
|  | Chairbound/Bedbound |  | 2.0 | (1.7, | 2.3) | 2.3 | (1.9, | 2.8) |  | 0.3 |  |
|  | Eyesight impairment |  | 8.5 | (7.9, | 9.1) | 6.6 | (5.9, | 7.3) |  | -1.9 |  |
|  | Hearing difficulties |  | 17.2 | (16.3, | 18.0) | 17.4 | (16.5, | 18.3) |  | 0.2 |  |
|  | Poor cognition (MMSE <24) |  | 23.0 | (22.0, | 24.0) | 16.3 | (15.3, | 17.2) |  | -6.7 |  |
|  | Poor self-reported health |  | 6.8 | (6.3, | 7.4) | 6.4 | (5.8, | 7.1) |  | -0.4 |  |
|  |  |  |  |  |  |  |  |  |  |  |  |
| **IADLs/ADLs**ᵇ | |  |  |  |  |  |  |  |  |  |  |
|  | Climb stairs |  | 37.7 | (36.6, | 38.8) | 37.5 | (36.3, | 38.7) |  | -0.2 |  |
|  | Cook a hot meal |  | 16.9 | (16.0, | 17.7) | 18.3 | (17.3, | 19.2) |  | 1.4 |  |
|  | Do heavy housework |  | 38.0 | (36.8, | 39.0) | 46.7 | (45.5, | 47.8) |  | 8.7 |  |
|  | Get on a bus |  | 28.8 | (27.8, | 29.8) | 25.7 | (24.6, | 26.8) |  | -3.1 |  |
|  | Reach an overhead shelf |  | 29.8 | (28.7, | 30.8) | 29.5 | (28.4, | 30.6) |  | -0.3 |  |
|  | Shop and carry bags |  | 47.2 | (46.1, | 48.3) | 50.4 | (49.3, | 51.6) |  | 3.2 |  |
|  | Cut own toenails |  | 43.7 | (42.6, | 44.9) | 49.5 | (48.3, | 50.7) |  | 5.8 |  |
|  | Put on own shoes or socks |  | 13.6 | (12.8, | 14.4) | 22.3 | (21.3, | 23.3) |  | 8.7 |  |
|  | Take a bath |  | 23.6 | (22.6, | 24.6) | 22.8 | (21.7, | 23.8) |  | -0.8 |  |
|  | Tie a knot |  | 9.6 | (8.9, | 10.3) | 12.0 | (11.2, | 12.9) |  | 2.4 |  |
|  |  |  |  |  |  |  |  |  |  |  |  |

ᵅ Depression is ever been diagnosed with depression by a doctor

ᵇ Inability to perform (Instrumental) Activities of Daily Living (ADLs/IADLs).

**Appendix 2- Mortality models for three analysis methods**

|  | **Model A**ᵅ | | |  | **Model B**ᵇ | | |  | **Model C**ᶜ | | |  | **Model D**ᵈ | | |  | **Final model**ᵉ | | |
| --- | --- | --- | --- | --- | --- | --- | --- | --- | --- | --- | --- | --- | --- | --- | --- | --- | --- | --- | --- |
| **1) Complete case analysis** | |  |  |  |  |  |  |  |  |  |  |  |  |  |  |  |  |  |  |
| Frailty Index | 6.53 | [5.02, | 8.05] |  | 4.12 | [2.46, | 5.76] |  | 4.15 | [2.50, | 5.79] |  | 4.20 | [2.55, | 5.85] |  | 4.22 | [2.57, | 5.87] |
| Frailty Index squared | -2.11 | [-4.80, | 0.57] |  | 0.75 | [-2.09, | 3.60] |  | 0.69 | [-2.15, | 3.53] |  | 0.61 | [-2.24, | 3.46] |  | 0.57 | [-2.26, | 3.41] |
| Study | -0.38 | [-0.51, | -0.24] |  | -0.48 | [-0.62, | -0.35] |  | -0.69 | [-0.88, | -0.50] |  | -3.01 | [-4.48, | -1.55] |  | -2.85 | [-4.31, | -1.38] |
| Sex |  |  | . |  | -0.67 | [-0.82, | -0.53] |  | -0.90 | [-1.08, | -0.72] |  | -0.67 | [-0.81, | -0.52] |  | -0.84 | [-1.02, | -0.67] |
| Age |  |  | . |  | 0.08 | [0.07, | 0.09] |  | 0.08 | [0.07, | 0.09] |  | 0.06 | [0.05, | 0.07] |  | 0.06 | [0.05, | 0.07] |
| Study by sex interaction | |  | . |  |  |  | . |  | 0.39 | [0.12, | 0.66] |  |  |  | . |  | 0.31 | [0.05, | 0.58] |
| Study by age interaction | |  | . |  |  |  | . |  |  |  | . |  | 0.03 | [0.01, | 0.05] |  | 0.03 | [0.01, | 0.05] |
| Constant | -3.50 | [-3.70, | -3.31] |  | -8.77 | [-9.53, | -7.99] |  | -8.67 | [-9.45, | -7.90] |  | -7.28 | [-8.21, | -6.37] |  | -7.40 | [-8.32, | -6.47] |
|  |  |  |  |  |  |  |  |  |  |  |  |  |  |  |  |  |  |  |  |
| AUC | 0.73 |  |  |  | 0.77 |  |  |  | 0.77 |  |  |  | 0.77 |  |  |  | 0.77 |  |  |
|  |  |  |  |  |  |  |  |  |  |  |  |  |  |  |  |  |  |  |  |
| **2) ≥ 29 items answered** |  |  |  |  |  |  |  |  |  |  |  |  |  |  |  |  |  |  |  |
| Frailty Index | 6.96 | [5.48, | 8.44] |  | 4.53 | [2.92, | 6.09] |  | 4.58 | [2.97, | 6.18] |  | 4.62 | [3.01, | 6.23] |  | 4.65 | [3.04, | 6.25] |
| Frailty Index squared | -2.68 | [-5.26, | -0.10] |  | 0.12 | [-2.62, | 2.86] |  | 0.03 | [-2.70, | 2.76] |  | -0.02 | [-2.76, | 2.72] |  | -0.08 | [-2.81, | 2.65] |
| Study | -0.34 | [-0.47, | -0.22] |  | -0.45 | [-0.58, | -0.32] |  | -0.70 | [-0.89, | -0.52] |  | -3.17 | [-4.59, | -1.74] |  | -2.95 | [-4.38, | -1.53] |
| Sex |  |  |  |  | -0.64 | [-0.78, | -0.50] |  | -0.92 | [-1.10, | -0.75] |  | -0.64 | [-0.78, | -0.50] |  | -0.86 | [-1.04, | -0.69] |
| Age |  |  |  |  | 0.08 | [0.07, | 0.09] |  | 0.08 | [0.07, | 0.09] |  | 0.06 | [0.05, | 0.07] |  | 0.06 | [0.05, | 0.07] |
| Study by sex interaction | |  |  |  |  |  | . |  | 0.47 | [0.21, | 0.73] |  |  |  | . |  | 0.38 | [0.12, | 0.65] |
| Study by age interaction | |  |  |  |  |  | . |  |  |  | . |  | 0.03 | [0.02 | 0.05] |  | 0.03 | [0.01, | 0.05] |
| Constant | -3.56 | [-3.75, | -3.37] |  | -8.86 | [-9.62, | -8.10] |  | -8.73 | [9.49, | -7.97] |  | -7.23 | [-8.12, | -6.35] |  | -7.37 | [-8.27 | 6.48] |
|  |  |  |  |  |  |  |  |  |  |  |  |  |  |  |  |  |  |  |  |
| AUC | 0.73 |  |  |  | 0.77 |  |  |  | 0.78 |  |  |  | 0.78 |  |  |  | 0.78 |  |  |
|  |  |  |  |  |  |  |  |  |  |  |  |  |  |  |  |  |  |  |  |

**Appendix 2- Mortality models for three analysis methods (continued)**

|  | **Model A**ᵅ | | |  | **Model B**ᵇ | | |  | **Model C**ᶜ | | |  | **Model D**ᵈ | | |  | **Final model**ᵉ | | |
| --- | --- | --- | --- | --- | --- | --- | --- | --- | --- | --- | --- | --- | --- | --- | --- | --- | --- | --- | --- |
| **3) MICE analysis** | |  |  |  |  |  |  |  |  |  |  |  |  |  |  |  |  |  |  |
| Frailty Index | 6.49 | [4.97, | 8.00] |  | 4.86 | [3.29, | 6.43] |  | 4.92 | [3.35, | 6.49] |  | 4.98 | [3.40, | 6.55] |  | 5.00 | [3.43, | 6.58] |
| Frailty Index squared | -2.03 | [-4.74, | 0.67] |  | -0.14 | [-2.76, | 2.47] |  | -0.23 | [-2.84, | 2.37] |  | -0.32 | [-2.94, | 2.29] |  | -0.37 | [-2.98, | 2.24] |
| Study | -0.38 | [-0.51, | -0.25] |  | -0.39 | [-0.51, | -0.27] |  | -0.66 | [-0.84, | -0.49] |  | -2.97 | [-4.29, | -1.65] |  | -2.73 | [-4.05, | -1.41] |
| Sex |  |  | . |  |  |  | . |  | 0.48 | [0.24, | 0.72] |  |  |  | . |  | -0.81 | [-0.97, | -0.65] |
| Age |  |  | . |  |  |  | . |  |  |  | . |  | 0.03 | [0.02, | 0.05] |  | 0.07 | [0.06, | 0.08] |
| Study by sex interaction | |  | . |  |  |  | . |  | 0.48 | [0.24, | 0.72] |  |  |  | . |  | 0.39 | [0.14, | 0.63] |
| Study by age interaction | |  | . |  |  |  | . |  |  |  | . |  | 0.03 | [0.02, | 0.05] |  | 0.03 | [0.01, | 0.04] |
| Constant | -3.49 | [-3.68, | -3.30] |  | -9.33 | [-10.05, | -8.62] |  | -9.19 | [-9.90, | -8.48] |  | -7.74 | [-8.56, | -6.91] |  | -7.90 | [-8.74, | -7.06] |
|  |  |  |  |  |  |  |  |  |  |  |  |  |  |  |  |  |  |  |  |
| AUC | 0.75 |  |  |  | 0.79 |  |  |  | 0.79 |  |  |  | 0.79 |  |  |  | 0.79 |  |  |
|  |  |  |  |  |  |  |  |  |  |  |  |  |  |  |  |  |  |  |  |

ᵅ Model A: Frailty and Cohort change (study)

ᵇ Model B: Frailty and Cohort change adjusted for sex and age differences

ᶜ Model C: Model B + interaction between sex and cohort change

ᵈ Model D: Model B + interaction between age and cohort change

ᵉ Final model: Frailty and Cohort change adjusted for sex and age differences, and interactions between cohort change and sex and cohort and age

Note: The mortality models for the three analysis methods: complete case analysis (8.4% missing), inclusion of those who answered 29 items or more (5.1% missing) and multiple imputation analysis (with 10 iterations) using chained equations (all missing data imputed). The coefficient (β) for each predictor from the logistic regression model and its 95% Confidence Interval are also shown. The exponential of these coefficients (exp(β)) correspond to the odds ratio of the covariate’s effect. Negative coefficients will result to ORs between 0 and 1, showing a protective effect against mortality. Positive coefficients correspond to ORs over 1, showing greater odds of mortality. Frailty index was modelled as the proportion of deficits present. The reference category for study was CFAS I and the reference gender group was men. The area under the receiver operating characteristic curve (ROC-AUC) is also denoted as a measure of model discrimination in predicting 2-year mortality.

**Appendix 3- A frequency distribution of the frailty index in CFAS I and II.**

Note: The frequencies were obtained using non-response weights in a multiple imputation framework

**Appendix 4 -Descriptive statistics of the frailty index and number of deaths by age bands and gender, for individuals aged 65 and over, Cambridgeshire, Newcastle and Nottingham, United Kingdom, 1991 (CFAS I) and 2011 (CFAS II).**

| Age Group | CFAS I | | | | CFAS II | | | |
| --- | --- | --- | --- | --- | --- | --- | --- | --- |
|  | Total | Deaths | Frailty | | Total | Deaths | Frailty | |
|  | N | N | Mean | Median (IQR) | N | N | Mean | Median (IQR) |
| Overall |  |  |  |  |  |  |  |  |
| 65-69 | 1981 | 83 | 0.12 | 0.07 (0.03, 0.17) | 1939 | 52 | 0.12 | 0.07 (0.03, 0.17) |
| 70-74 | 1776 | 110 | 0.14 | 0.10 (0.03, 0.20) | 1873 | 75 | 0.15 | 0.10 (0.07, 0.23) |
| 75-79 | 1725 | 195 | 0.17 | 0.13 (0.07, 0.27) | 1624 | 96 | 0.19 | 0.17 (0.07, 0.27) |
| 80-84 | 1308 | 221 | 0.23 | 0.20 (0.10, 0.33) | 1278 | 160 | 0.23 | 0.20 (0.10, 0.33) |
| 85+ | 845 | 210 | 0.3 | 0.30 (0.20, 0.40) | 1048 | 260 | 0.31 | 0.33 (0.20, 0.43) |
| All ages | 7635 | 819 | 0.18 | 0.13 (0.07, 0.27) | 7762 | 643 | 0.19 | 0.17 (0.07, 0.30) |
| Men |  |  |  |  |  |  |  |  |
| 65-69 | 915 | 57 | 0.11 | 0.07 (0.03, 0.13) | 968 | 29 | 0.11 | 0.07 (0.03, 0.13) |
| 70-74 | 780 | 67 | 0.12 | 0.10 (0.03, 0.17) | 902 | 47 | 0.14 | 0.10 (0.03, 0.20) |
| 75-79 | 696 | 110 | 0.16 | 0.10 (0.07, 0.23) | 758 | 52 | 0.16 | 0.13 (0.07, 0.23) |
| 80-84 | 449 | 93 | 0.2 | 0.17 (0.10, 0.30) | 542 | 74 | 0.21 | 0.17 (0.07, 0.30) |
| 85+ | 205 | 59 | 0.26 | 0.27 (0.13, 0.37) | 364 | 92 | 0.26 | 0.27 (0.16, 0.40) |
| All ages | 3045 | 386 | 0.15 | 0.10 (0.03, 0.20) | 3534 | 294 | 0.16 | 0.10 (0.07, 0.23) |
| Women |  |  |  |  |  |  |  |  |
| 65-69 | 1066 | 26 | 0.13 | 0.10 (0.03, 0.17) | 971 | 23 | 0.12 | 0.07 (0.03, 0.17) |
| 70-74 | 996 | 43 | 0.15 | 0.10 (0.03, 0.23) | 971 | 28 | 0.17 | 0.13 (0.06, 0.24) |
| 75-79 | 1029 | 85 | 0.2 | 0.17 (0.07, 0.27) | 866 | 44 | 0.21 | 0.17 (0.10, 0.30) |
| 80-84 | 859 | 128 | 0.25 | 0.23 (0.13, 0.36) | 736 | 86 | 0.26 | 0.23 (0.13, 0.37) |
| 85+ | 640 | 151 | 0.31 | 0.33 (0.20, 0.40) | 684 | 168 | 0.33 | 0.33 (0.23, 0.43) |
| All ages | 4590 | 433 | 0.2 | 0.17 (0.07, 0.30) | 4228 | 349 | 0.21 | 0.20 (0.07, 0.33) |
|  |  |  |  |  |  |  |  |  |

**Appendix 5: Predicted frailty index in a model adjusting for age, study and gender.**

**
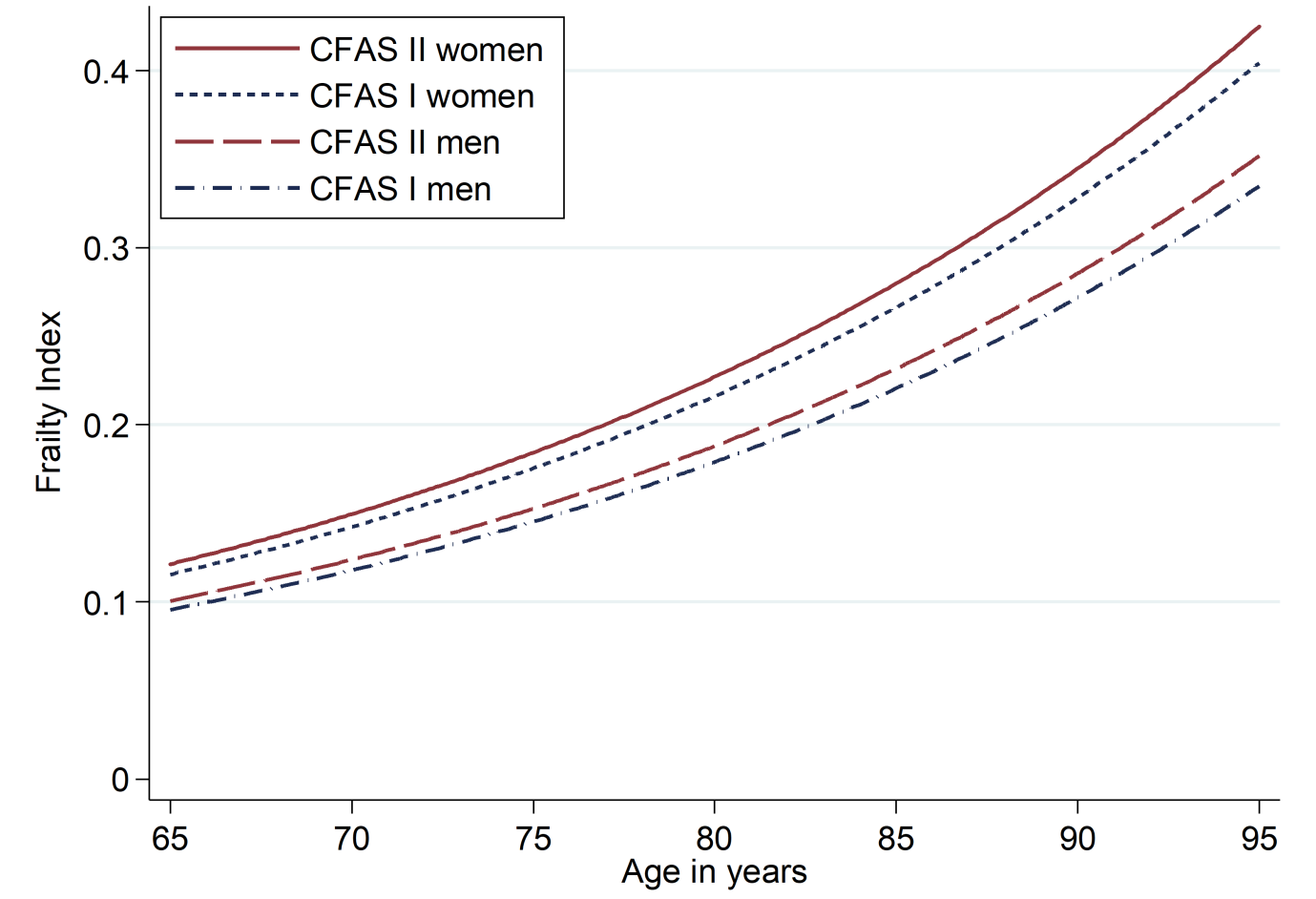
**

**Appendix 6: The relationship between frailty and mortality for different frailty index (FI) scores**

|  | **Effect of a 0.1 increase in FI** | | **Effect compared to FI=0** | |
| --- | --- | --- | --- | --- |
|  |  |  |  |  |
| **Frailty Index (FI)** | **Ref FI** | **Odds Ratio (95%CI)** | **Ref FI** | **Odds Ratio (95%CI)** |
|  |  |  |  |  |
| **Model A**ᵅ |  |  |  |  |
| **0.1** | 0 | 2.08 (1.82, 2.33) | 0 | 2.08 ( 1.82, 2.33) |
| **0.2** | 0.1 | 1.96 (1.80, 2.11) | 0 | 4.06 ( 3.26, 4.87) |
| **0.3** | 0.2 | 1.84 (1.76, 1.92) | 0 | 7.48 ( 5.75, 9.22) |
| **0.4** | 0.3 | 1.74 (1.65, 1.83) | 0 | 12.99 ( 9.99, 15.99) |
| **0.5** | 0.4 | 1.64 (1.49, 1.78) | 0 | 21.24 (16.60, 25.88) |
| **0.6** | 0.5 | 1.54 (1.33, 1.75) | 0 | 32.72 (24.65, 40.79) |
| **0.7** | 0.6 | 1.45 (1.18, 1.72) | 0 | 47.49 (30.26, 64.72) |
| **Model Bᵇ** |  |  |  |  |
| **0.1** | 0 | 1.62 (1.41, 1.84) | 0 | 1.62 ( 1.41, 1.84) |
| **0.2** | 0.1 | 1.62 (1.48, 1.76) | 0 | 2.63 ( 2.06, 3.20) |
| **0.3** | 0.2 | 1.61 (1.54, 1.69) | 0 | 4.25 ( 3.17, 5.32) |
| **0.4** | 0.3 | 1.61 (1.52, 1.70) | 0 | 6.84 ( 5.09, 8.59) |
| **0.5** | 0.4 | 1.61 (1.45, 1.76) | 0 | 10.97 ( 8.33, 13.62) |
| **0.6** | 0.5 | 1.60 (1.37, 1.83) | 0 | 17.57 (12.91, 22.24) |
| **0.7** | 0.6 | 1.60 (1.29, 1.91) | 0 | 28.05 (17.38, 38.73) |
| **Final modelᶜ** |  |  |  |  |
| **0.1** | 0 | 1.64 (1.43, 1.86) | 0 | 1.64 ( 1.43, 1.86) |
| **0.2** | 0.1 | 1.63 (1.49, 1.77) | 0 | 2.68 ( 2.10, 3.26) |
| **0.3** | 0.2 | 1.62 (1.54, 1.70) | 0 | 4.34 ( 3.23, 5.45) |
| **0.4** | 0.3 | 1.61 (1.52, 1.70) | 0 | 6.98 ( 5.19, 8.77) |
| **0.5** | 0.4 | 1.60 (1.44, 1.75) | 0 | 11.14 ( 8.42, 13.85) |
| **0.6** | 0.5 | 1.58 (1.36, 1.81) | 0 | 17.64 (12.92, 22.36) |
| **0.7** | 0.6 | 1.57 (1.27, 1.88) | 0 | 27.74 (17.17, 38.31) |
|  |  |  |  |  |

ᵅ Model A: Frailty and Cohort change (study)

ᵇ Model B: Frailty and Cohort change adjusted for sex and age differences

ᶜFinal model: Frailty and Cohort change adjusted for sex and age differences, and interactions between cohort change and sex and cohort and age

Note: Odds ratios and corresponding 95%Confidence Intervals are denoted for each comparison. All models were adjusted for the non-linear effect of frailty.

**Appendix 7- Predicted mortality (%) by frailty for ages 65 (A), 75 (B), and 85 (C) shown for CFAS I men, CFAS II men, CFAS I women, and CFAS II women. The predicted lines were obtained from the final model which includes the study interactions by age and gender**


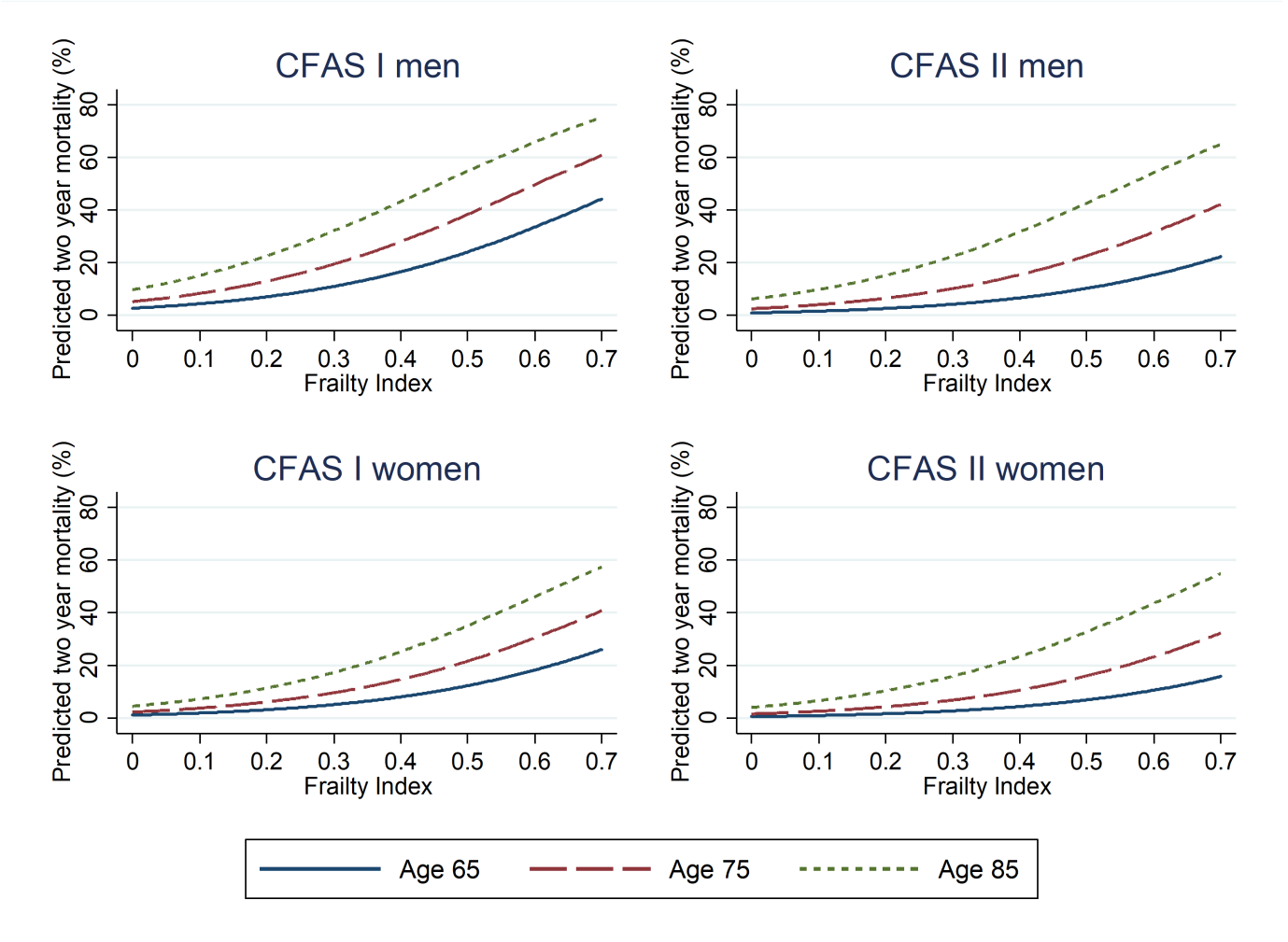


**Appendix 8- Predicted probabilities (as a percentage) of two year mortality for different numbers of deficits present, by age and sex.**

|  | **Predicted probability of 2-year mortality as a percentage (%)** | | | | | | | | | | | | | | |
| --- | --- | --- | --- | --- | --- | --- | --- | --- | --- | --- | --- | --- | --- | --- | --- |
|  | **Men** | | | | | | |  | **Women** | | | | | | |
| **Frailty** | **CFAS I** | | |  | **CFAS II** | | |  | **CFAS I** | | |  | **CFAS II** | | |
| **Age** | **65** | **75** | **85** |  | **65** | **75** | **85** |  | **65** | **75** | **85** |  | **65** | **75** | **85** |
| **None** | 2.8 | 5.3 | 9.9 |  | 1.0 | 2.6 | 6.3 |  | 1.3 | 2.4 | 4.6 |  | 0.7 | 1.7 | 4.2 |
| **1 item** | 3.3 | 6.2 | 11.4 |  | 1.2 | 3.0 | 7.3 |  | 1.5 | 2.9 | 5.4 |  | 0.8 | 2.0 | 4.9 |
| **2 items** | 3.8 | 7.2 | 13.2 |  | 1.4 | 3.5 | 8.5 |  | 1.7 | 3.4 | 6.3 |  | 0.9 | 2.3 | 5.8 |
| **3 items** | 4.5 | 8.4 | 15.2 |  | 1.7 | 4.2 | 9.9 |  | 2.1 | 3.9 | 7.4 |  | 1.1 | 2.8 | 6.7 |
| **4 items** | 5.3 | 9.8 | 17.5 |  | 2.0 | 4.9 | 11.5 |  | 2.4 | 4.6 | 8.6 |  | 1.3 | 3.2 | 7.8 |
| **5 items** | 6.1 | 11.3 | 19.9 |  | 2.3 | 5.7 | 13.3 |  | 2.8 | 5.4 | 10.0 |  | 1.5 | 3.8 | 9.1 |
| **6 items** | 7.1 | 13.0 | 22.7 |  | 2.7 | 6.6 | 15.2 |  | 3.3 | 6.3 | 11.5 |  | 1.8 | 4.4 | 10.5 |
| **7 items** | 8.3 | 15.0 | 25.6 |  | 3.2 | 7.7 | 17.4 |  | 3.9 | 7.3 | 13.3 |  | 2.1 | 5.2 | 12.1 |
| **8 items** | 9.6 | 17.2 | 28.8 |  | 3.7 | 8.9 | 19.9 |  | 4.5 | 8.4 | 15.2 |  | 2.5 | 6.0 | 14.0 |
| **9 items** | 11.1 | 19.5 | 32.2 |  | 4.6 | 10.3 | 22.4 |  | 5.2 | 9.7 | 17.4 |  | 2.9 | 7.0 | 16.0 |
| **10 items** | 12.7 | 22.2 | 35.7 |  | 5.0 | 11.8 | 25.4 |  | 6.1 | 11.2 | 19.8 |  | 3.3 | 8.1 | 18.3 |
| **11 items** | 14.6 | 25.0 | 39.4 |  | 5.8 | 13.6 | 28.5 |  | 7.1 | 12.9 | 22.5 |  | 3.9 | 9.3 | 20.7 |
| **12 items** | 16.7 | 28.1 | 43.2 |  | 6.8 | 15.5 | 31.9 |  | 8.2 | 14.8 | 25.3 |  | 4.8 | 10.8 | 23.4 |
| **13 items** | 19.0 | 31.4 | 47.1 |  | 8.3 | 17.7 | 35.4 |  | 9.4 | 16.9 | 28.4 |  | 5.6 | 12.4 | 26.4 |
| **14 items** | 21.5 | 34.8 | 51.0 |  | 9.6 | 20.1 | 39.0 |  | 10.8 | 19.2 | 31.6 |  | 6.5 | 14.1 | 29.5 |
| **15 items** | 24.2 | 38.4 | 54.8 |  | 10.4 | 22.7 | 42.7 |  | 12.4 | 21.6 | 35.1 |  | 7.5 | 16.1 | 32.8 |
| **16 items** | 27.1 | 42.1 | 58.5 |  | 12.6 | 25.5 | 46.6 |  | 14.1 | 24.4 | 38.7 |  | 8.1 | 18.4 | 36.3 |
| **17 items** | 30.3 | 45.9 | 62.4 |  | 14.4 | 28.6 | 50.5 |  | 16.4 | 27.4 | 42.4 |  | 9.9 | 22.1 | 39.9 |
| **18 items** | 33.6 | 49.7 | 65.7 |  | 16.4 | 31.7 | 54.0 |  | 18.3 | 30.5 | 46.1 |  | 11.4 | 24.8 | 43.8 |
| **19 items** | 37.1 | 53.5 | 69.2 |  | 18.6 | 37.0 | 60.1 |  | 20.7 | 33.8 | 49.9 |  | 13.0 | 27.7 | 47.2 |
| **20 items** | 40.6 | 57.2 | 72.7 |  | 21.0 | 40.5 | 63.6 |  | 23.3 | 37.3 | 53.7 |  | 14.8 | 30.9 | 51.6 |

**Appendix 9-AUC values for the prediction of death up to two years shown separately for CFAS I and II**

| **MODEL** | **CFAS I** | **CFAS II** |
| --- | --- | --- |
| Age+Sex | 0.71 | 0.76 |
| Frailty | 0.72 | 0.77 |
| Frailty+Sex | 0.73 | 0.77 |
| Frailty+Age | 0.75 | 0.80 |
| Frailty+Sex+Age | 0.76 | 0.81 |

Note: The models that included frailty as a covariate also included the non-linear effect of frailty

**Appendix 10- Sensitivity analysis: (a) survival analysis and (b) removal of 3 readily diagnosed items**

|  | **Adjusted model** | | | **Stratified analysis** | | | | | | |
| --- | --- | --- | --- | --- | --- | --- | --- | --- | --- | --- |
|  |  |  |  | **CFAS I** | | |  | **CFAS II** | | |
|  | **Coefficient [95% CI]** | | | **Coefficient [95% CI]** | | |  | **Coefficient [95% CI]** | | |
| **(a) Cox regression** |  |  |  |  |  |  |  |  |  |  |
| Frailty Index | 5.22 | [ 3.79, | 6.64] | 4.44 | [ 2.62, | 6.26] |  | 5.91 | [ 3.74, | 8.08] |
| Frailty Index squared | -1.42 | [-3.68, | 0.84] | -0.64 | [-3.71, | 2.43] |  | -2.18 | [-5.47, | 1.11] |
| Study | -3.03 | [-4.20, | -1.87] |  |  |  |  |  |  |  |
| Sex | -0.73 | [-0.87, | -0.58] | -0.71 | [-0.86, | -0.57] |  | -0.39 | [-0.56, | -0.22] |
| Age | 0.06 | [ 0.05, | 0.07] | 0.06 | [ 0.05, | 0.07] |  | 0.09 | [ 0.07, | 0.10] |
| Study by sex interaction | 0.35 | [ 0.13, | 0.57] |  |  |  |  |  |  |  |
| Study by age interaction | 0.03 | [ 0.02, | 0.04] |  |  |  |  |  |  |  |
|  |  |  |  |  |  |  |  |  |  |  |
| **(b) removing 3 items: hypertension, thyroid problems and diabetes** |  |  |  |  |  |  |  |  |  |  |
| Frailty Index | 4.67 | [ 3.22, | 6.13] | 3.71 | [ 1.91, | 5.52] |  | 5.41 | [ 3.24, | 7.59] |
| Frailty Index squared | -0.19 | [-2.54, | 2.17] | 0.80 | [-2.19, | 3.79] |  | -0.98 | [-4.45, | 2.49] |
| Study | -2.68 | [-3.99, | -1.36] |  |  |  |  |  |  |  |
| Sex | -0.79 | [-0.96, | -0.63] | -0.77 | [-0.93, | -0.61] |  | -0.42 | [-0.61, | -0.23] |
| Age | 0.06 | [ 0.05, | 0.07] | 0.07 | [ 0.06, | 0.08] |  | 0.09 | [ 0.07, | 0.10] |
| Study by sex interaction | 0.38 | [ 0.14, | 0.63] |  |  |  |  |  |  |  |
| Study by age interaction | 0.03 | [ 0.01, | 0.04] |  |  |  |  |  |  |  |
|  |  |  |  |  |  |  |  |  |  |  |
| **(c) removing 2 items: cognitive impairment and depression** |  |  |  |  |  |  |  |  |  |  |
| Frailty Index | 4.70 | [ 3.17, | 6.24] | 4.23 | [ 2.33, | 6.12] |  | 5.19 | [ 2.84, | 7.54] |
| Frailty Index squared | -0.32 | [-2.83, | 2.19] | 0.11 | [-3.15, | 3.36] |  | -0.85 | [-4.52, | 2.82] |
| Study | -2.70 | [-4.03, | -1.38] |  |  |  |  |  |  |  |
| Sex | -0.79 | [-0.95, | -0.63] | -0.78 | [-0.94, | -0.61] |  | -0.41 | [-0.60, | -0.22] |
| Age | 0.07 | [ 0.06, | 0.08] | 0.07 | [ 0.06, | 0.08] |  | 0.09 | [ 0.08, | 0.11] |
| Study by sex interaction | 0.38 | [ 0.14, | 0.63] |  |  |  |  |  |  |  |
| Study by age interaction | 0.03 | [ 0.01, | 0.04] |  |  |  |  |  |  |  |
|  |  |  |  |  |  |  |  |  |  |  |

(a) Survival analysis and was used as a sensitivity analysis. The coefficient (β) for each covariate from the Cox regression model and its 95% Confidence Interval are shown. The exponential of these coefficients (exp(β)) correspond to the hazard ratio(HR) of the covariate’s effect. Negative coefficients will result to HRs between 0 and 1, showing a protective effect against mortality. Positive coefficients correspond to HRs over 1, showing greater risk of death.

(b) Another sensitivity analysis included the removal of 3 items from the frailty index which are now more readily diagnosed (hypertension, thyroid problems and diabetes). The coefficients and 95% CIs shown are from the logistic regression model. The exponential of those correspond to the Odds Ratios (ORs) of the covariate's effect.

(c) As a further sensitivity analysis, cognitive impairment and depression, which are markers of non-participation, were removed from the frailty index. Coefficients and 95% CIs are interpreted as in (b).

Frailty index was modelled as the proportion of deficits present. The reference category for study was CFAS I and the reference gender group was men. This analysis used non-response weights on the imputed data. Results are shown for the final (adjusted model) and for the models stratified by study.
