# Supplementary material for: Is frailty a stable predictor of mortality across time? Evidence from the Cognitive Function and Ageing Studies
Source: Age Ageing. 2018 Jun 16;47(5):721–7. doi: 10.1093/ageing/afy077 (PMC6108394; doi:10.1093/ageing/afy077)
Supplement: Supplementary Data [file afy077_aa-17-1121-file004.docx]

**REFERENCES (FULL REFERENCE LIST)**

1. Gonzalez EL, Johansson S, Wallander MA, Rodriguez LA. Trends in the prevalence and incidence of diabetes in the UK: 1996-2005. J Epidemiol Community Health. 2009;63(4):332-6.

2. Blachier M, Leleu H, Peck-Radosavljevic M, Valla D-C, Roudot-Thoraval F. The burden of liver disease in Europe: A review of available epidemiological data. J Hepatol. 2013;58(3):593-608.

3. Jagger C, Matthews FE, Wohland P, Fouweather T, Stephan BCM, Robinson L, et al. A comparison of health expectancies over two decades in England: results of the Cognitive Function and Ageing Study I and II. Lancet. 2016;387(10020):779-86.

4. Clegg A, Young J, Iliffe S, Rikkert MO, Rockwood K. Frailty in elderly people. Lancet. 2013;381(9868):752-62.

5. Mitnitski AB, Mogilner AJ, Rockwood K. Accumulation of deficits as a proxy measure of aging. TheScientificWorldJournal. 2001;1:323-36.

6. Fried LP, Tangen CM, Walston J, Newman AB, Hirsch C, Gottdiener J, et al. Frailty in older adults: evidence for a phenotype. J Gerontol A Biol Sci Med Sci. 2001;56(3):M146-56.

7. Dent E, Kowal P, Hoogendijk EO. Frailty measurement in research and clinical practice: A review. Eur J Intern Med. 2016 Jun;31:3-10.

8. Adachi JD, Ioannidis G, Pickard L, Kennedy C, Papaioannou A, Thabane L. Frailty index of deficit accumulation and falls: data from the Global Longitudinal Study of Osteoporosis in Women (GLOW) Hamilton cohort. BMC Musculoskelet Disord. 2014;15:185.

9. Searle SD, Mitnitski A, Gahbauer EA, Gill TM, Rockwood K. A standard procedure for creating a frailty index. BMC Geriatr. 2008;8:24.

10. Goggins WB, Woo J, Sham A, Ho SC. Frailty index as a measure of biological age in a Chinese population. J Gerontol A Biol Sci Med Sci. 2005;60(8):1046-51.

11. Kulminski A, Ukraintseva SV, Akushevich I, Arbeev KG, Land K, Yashin AI. Accelerated accumulation of health deficits as a characteristic of aging. Exp Gerontol. 2007;42(10):963-70.

12. Romero-Ortuno R, Kenny RA. The frailty index in Europeans: Association with age and mortality. Age Ageing. 2012;41(5):684-9.

13. Widagdo IS, Pratt N, Russell M, Roughead EE. Predictive performance of four frailty measures in an older Australian population. Age Ageing. 2015;44(6):967-72.

14. Malmstrom TK, Miller DK, Morley JE. A comparison of four frailty models. J Am Geriatr Soc. 2014;62(4):721-6.

15. Clegg A, Bates C, Young J, Ryan R, Nichols L, Ann Teale E, et al. Development and validation of an electronic frailty index using routine primary care electronic health record data. Age Ageing. 2016;45(3):353-60.

16. Martin BJ, Valiyaparambath N, Gray J. Patient outcomes using revised guidance NHS continuing care eligibility criteria, Scotland (CEL 2008). Scott Med J. 2013;58(1):16-9.

17. Rockwood K, Song X, Mitnitski A. Changes in relative fitness and frailty across the adult lifespan: Evidence from the Canadian National Population Health Survey. CMAJ. 2011;183(8):E487-E94.

18. Haque S, Reeves MJ, Ofner S, Ordin D, Williams LS, Bravata D. Frailty independently predicts poor outcomes but not quality of care: An analysis of VA ischemic stroke patients. International Stroke Conference; New Orleans, Louisiana: American Heart Association; 2012.

19. Dent E, Hoon E, Karnon J, Newbury J, Kitson A, Beilby J. Frailty and health service use in rural South Australia. Arch Gerontol Geriatr. 2016 01 Jan;62:53-8.

20. Shi J, Song X, Yu P, Tang Z, Mitnitski A, Fang X, et al. Analysis of frailty and survival from late middle age in the Beijing Longitudinal Study of Aging. BMC Geriatr. 2011;11:17.

21. Saum KU, Dieffenbach AK, Muller H, Holleczek B, Hauer K, Brenner H. Frailty prevalence and 10-year survival in community-dwelling older adults: Results from the ESTHER cohort study. Eur J Epidemiol. 2014;29(3):171-9.

22. Wang C, Song X, Mitnitski A, Fang X, Tang Z, Yu P, et al. Effect of health protective factors on health deficit accumulation and mortality risk in older adults in the Beijing longitudinal study of aging. J Am Geriatr Soc. 2014;62(5):821-8.

23. Backman K, Joas E, Falk H, Mitnitski A, Rockwood K, Skoog I. Changes in the Lethality of Frailty Over 30 Years: Evidence From Two Cohorts of 70-Year-Olds in Gothenburg Sweden. J Gerontol A Biol Sci Med Sci. 2016.

24. Matthews FE, Arthur A, Barnes LE, Bond J, Jagger C, Robinson L, et al. A two-decade comparison of prevalence of dementia in individuals aged 65 years and older from three geographical areas of England: results of the Cognitive Function and Ageing Study I and II. Lancet. 2013;382(9902):1405-12.

25. Gao L, Green E, Barnes LE, Brayne C, Matthews FE, Robinson L, et al. Changing non-participation in epidemiological studies of older people: evidence from the Cognitive Function and Ageing Study I and II. Age Ageing. 2015;44(5):867-73.

26. Mitnitski A, Rockwood K. The rate of aging: the rate of deficit accumulation does not change over the adult life span. Biogerontology. 2016;17(1):199-204.

27. López-Otín C, Blasco MA, Partridge L, Serrano M, Kroemer G. The Hallmarks of Aging. Cell.153(6):1194-217.

28. Theou O, O'Connell MDL, King-Kallimanis BL, O'Halloran AM, Rockwood K, Kenny RA. Measuring frailty using self-report and test-based health measures. Age Ageing. 2015;44(3):471-7.

29. White IR, Carlin JB. Bias and efficiency of multiple imputation compared with complete-case analysis for missing covariate values. Stat Med. 2010;29(28):2920-31.

30. McCaul KA, Almeida OP, Norman PE, Yeap BB, Hankey GJ, Golledge J, et al. How many older people are frail? Using multiple imputation to investigate frailty in the population. J Am Med Dir Assoc. 2015;16(5):439.e1-.e7.

31. Mitnitski A, Song X, Skoog I, Broe GA, Cox JL, Grunfeld E, et al. Relative fitness and frailty of elderly men and women in developed countries and their relationship with mortality. J Am Geriatr Soc. 2005;53(12):2184-9.

32. Ma L, Zhang L, Tang Z, Sun F, Diao L, Wang J, et al. Use of the frailty index in evaluating the prognosis of older people in Beijing: A cohort study with an 8-year follow-up. Arch Gerontol Geriatr. 2016;64:172-7.

33. Theou O, Brothers TD, Pena FG, Mitnitski A, Rockwood K. Identifying common characteristics of frailty across seven scales. J Am Geriatr Soc. 2014;62(5):901-6.

34. Hubbard RE, Rockwood K. Frailty in older women. Maturitas. 2011;69(3):203-7.

35. Drubbel I, de Wit NJ, Bleijenberg N, Eijkemans RJ, Schuurmans MJ, Numans ME. Prediction of adverse health outcomes in older people using a frailty index based on routine primary care data. J Gerontol A Biol Sci Med Sci. 2013;68(3):301-8.

36. Armstrong JJ, Mitnitski A, Launer LJ, White LR, Rockwood K. Frailty in the Honolulu-Asia Aging Study: deficit accumulation in a male cohort followed to 90% mortality. J Gerontol A Biol Sci Med Sci. [Research Support, Non-U.S. Gov't]. 2015;70(1):125-31.

37. Shi J, Yang Z, Song X, Yu P, Fang X, Tang Z, et al. Sex differences in the limit to deficit accumulation in late middle-aged and older Chinese people: results from the Beijing Longitudinal Study of Aging. J Gerontol A Biol Sci Med Sci. 2014;69(6):702-9.

38. Gordon EH, Peel NM, Samanta M, Theou O, Howlett SE, Hubbard RE. Sex differences in frailty: A systematic review and meta-analysis. Exp Gerontol. 2017;89:30-40.

39. Robinson L. Present and future configuration of health and social care services to enhance robustness in older age. London: The Stationery Office, 2014.
